# Gamma-Glutamyl Transpeptidase-to-Platelet Ratio Predicts Significant Liver Fibrosis of Chronic Hepatitis B Patients in China

**DOI:** 10.1155/2017/7089702

**Published:** 2017-07-31

**Authors:** Tianyi Ren, Huan Wang, Ruihong Wu, Junqi Niu

**Affiliations:** ^1^Department of Hepatology, The First Hospital of Jilin University, Changchun, Jilin 130021, China; ^2^Jilin Province Key Laboratory of Infectious Disease, Laboratory of Molecular Virology, Changchun 130021, China; ^3^Key Laboratory of Zoonosis Research, Ministry Education, Changchun, Jilin 130021, China

## Abstract

**Background and Aims:**

We want to investigate whether a novel noninvasive marker is suitable for Chinese CHB patients.

**Methods:**

A total of 160 treatment-naïve CHB patients who underwent liver biopsy were enrolled in our study, and we assessed the diagnostic accuracies of GPR, aspartate transaminase-to-platelet ratio index (APRI), and the fibrosis index based on 4 factors (FIB-4) in them.

**Results:**

Of these 160 CHB patients, the numbers of F0, F1, F2, F3, and F4 are 34 (21.3%), 62 (38.8%), 18 (11.3%), 24 (15%), and 22 (13.8%), respectively. The area under the receiver operating characteristic curves (AUROC) of GPR for fibrosis (0.77 versus 0.70, *P* = 0.03), significant fibrosis (0.70 versus 0.63, *P* = 0.02), and extensive fibrosis (0.71 versus 0.64, *P* = 0.02) were significantly higher than those of APRI. The AUROCs of GPR and Fib-4 for fibrosis (0.77 versus 0.75, *P* = 0.14), significant fibrosis (0.70 versus 0.70, *P* = 0.22), extensive fibrosis (0.71 versus 0.68, *P* = 0.13), and cirrhosis (0.64 versus 0.67, *P* = 0.24) were comparable.

**Conclusions:**

The GPR can be a routine laboratory marker to stage liver fibrosis in patients with CHB in China.

## 1. Introduction

Hepatitis B virus (HBV) infection threatens 350 million people worldwide, particularly in Eastern Asia. In China, HBV infection is moderately endemic. Persistent infection with HBV can evolve into cirrhosis and then into hepatocellular carcinoma (HCC), which is one of the most frequent cancers in China [1]. To reduce the disease burden of HBV infection, it may be critical to identify subjects with fibrosis or cirrhosis and treat them with antiviral therapy as soon as possible [2].

The recognized gold standard for diagnosing fibrosis and cirrhosis is liver biopsy (LB), which is not currently performed in all hospitals, considering the invasiveness, expensive procedure, and complications. Transient elastography (Fibroscan), for liver stiffness measurement, is increasingly valued as another important method for diagnosing fibrosis and cirrhosis because of its noninvasive feature, repeatability, and excellent efficacy [2, 3]. But a Fibroscan device is still so expensive (for machine and annual maintenance) that it could only be accessed in main hospitals or big cities in China. Thus, it is urgent to develop simple, cheap, and noninvasive fibrosis models for China and other developing countries with considerable HBV prevalence.

In recent years, the introduction of novel serum models for diagnosing fibrosis and cirrhosis has become a hot issue among relative researchers. In March 2015, the aspartate transaminase- (AST-) to-platelet ratio index (APRI) and fibrosis index based on 4 factors (Fib-4) were recommended by the first WHO guidelines on the prevention, care, and treatment of patients with chronic hepatitis B (CHB) as noninvasive tools to detect fibrosis and cirrhosis in resource-limited settings [4]. Although they both have advantages of comprising only two or three inexpensive laboratory tests, the APRI and Fib-4 have still faced other problems, such as low level of sensitivity and the lack of enough accuracy for diagnosing mild-to-moderate liver fibrosis. Under such circumstances, new models are in great need.

In June 2015, Lemoine et al. reported a novel noninvasive biomarker—the gamma-glutamyl transpeptidase (GGT)-to-platelet ratio (GPR)—to identify HBV-infected subjects with significant fibrosis or cirrhosis in West Africa. Their study enrolled 135 CHB patients in Gambia, West Africa, and they assessed its diagnostic accuracy in two external validation cohorts (80 patients from Senegal and 63 patients from France) [5]. The results show that GPR is more accurate than APRI and Fib-4 in West Africa, but not superior to APRI and Fib-4 in France [6]. However, in February 2016, Li et al. assessed the diagnostic accuracy of this new serum fibrosis model through a retrospective study in Shanghai, China, and 372 CHB patients who underwent liver biopsy and routine laboratory tests were included [7]. Their results show that GPR is less accurate than the APRI and comparable to Fib-4 in China [7]. Meanwhile, researchers from Hebei Province, China, carried out a prospective observational study to evaluate the diagnostic ability of this novel biomarker too. Their results showed that GPR was more reliable to predict fibrosis stage [8]. All 3 groups of scientists have done great works but their results differ. According to authors' conclusions, there needs to be more data to access this model's accuracy and to verify whether GPR deserves utilization in China or not [5, 7, 8]. So, using liver biopsy as the standard, we compared the diagnosing performance of GPR with that of APRI and Fib-4 in 160 CHB patients.

## 2. Methods

### 2.1. Patients

A total of 170 treatment-naïve CHB patients who underwent liver biopsy at the First Hospital of Jilin University, between January 2010 and May 2016, were retrospectively screened. We performed liver biopsies to these patients for fibrosis staging, inflammation grading, and differential diagnosis.

Other causes of liver disease have been excluded. We also excluded 10 patients for the deficiency of clinical data. This study was conducted in accordance with the Declaration of Helsinki and was approved by the First Hospital of Jilin University Ethics Committee.

### 2.2. Clinical and Laboratory Assessment

Demographics, liver biochemistry, hepatitis virus, and hematological parameters were collected and documented by blinded clinicians to prevent bias. Fasting venous blood samples were collected between 5:30 and 6:00 AM on the day of procedure. Liver biochemistry tests included AST, ALT, and GGT. Hematological analyses involved red blood cell (RBC), white blood cell (WBC), hemoglobin, platelets, mean platelet volume (MPV), mean corpuscular volume (MCV), and platelet distribution width (PDW).

### 2.3. Histological Assessment

As all biopsies were guided by the color Doppler ultrasonography under local anesthesia, a 16 G Tru-Cut needle was applied for the biopsy performance. The specimens were fixed in buffered formalin, embedded in paraffin, and stained with hematoxylin and eosin and Masson trichrome stain. A minimum of 1.5 cm of liver tissue containing at least 5 portal tracts was required for diagnosis by the histopathologists in our hospital, a tertiary referral teaching hospital in China. The pathological diagnosis of each sample was determined based on the METAVIR scoring system, and the fibrosis was classified into 5 stages: F0, no fibrosis; F1, expansion of portal zones; F2, expansion of most portal zones and occasional bridging; F3, expansion of most portal zones and marked bridging and occasional modules; and F4, cirrhosis.

### 2.4. Formulas

Consider the following:

(1) $$\begin{array}{c} GPR=\frac{\left(GGT\;\left(IU/L\right)/ULN\;of\;GGT\right)}{platelet\;count\;\left(10^{9}/L\right)}\times 100.\\ APRI=\frac{\left(AST\;\left(IU/L\right)/ULN\;of\;AST\right)}{platelet\;count\;\left(10^{9}/L\right)}\times 100.\\ Fib‐4=\frac{\left(age\;\left(years\right)\times AST\;\left(IU/L\right)\right)}{\left(platelet\;count\;\left(10^{9}/L\right)\times {\left(ALT\;\left(IU/L\right)\right)}^{1/2}\right)}. \end{array}$$

### 2.5. Statistical Analysis

Continuous variables were presented as medians (25th and 75th percentiles), while categorical variables were displayed as numbers and percentages. Mann–Whitney test and *t*-test were used for comparison of continuous variables between two groups, if necessary. We used the receiver operating characteristic (ROC) curve to estimate the diagnostic accuracies of established fibrosis models (APRI and Fib-4) and the new one (GPR). The sensitivity, specificity, positive predictive values, and negative predictive values and the area under the ROC curve (AUROC) of each test for fibrosis (≥F1), significant fibrosis (≥F2), extensive fibrosis (≥F3), and cirrhosis (F4) were obtained by comparing patients of F1–4 with that of F0, F2–4 with F0–1, F3–4 with F0–2, and F4 with F0–3, respectively. For APRI and Fib-4, predefined cut-offs were used (0.5 and 1.5 for APRI to distinguish F0–1 and F2–4, 1.0 and 2.0 for APRI to distinguish F0–3 and F4, and 1.45 and 3.25 for Fib-4 to distinguish F0–2 and F3–4) [5]. All *P* values provided are 2-sided and a *P* < 0.05 was considered statistically significant. All statistical analyses were carried out using the SPSS statistical software version 22.0 (SPSS Inc., Chicago, IL).

## 3. Results

Baseline characteristics of the study population are presented in Table 1. A total of 160 patients were enrolled in this study, of which 101 (63.13%) were men and 59 (36.87%) were women. The numbers of patients in F0, F1, F2, F3, and F4 stages are 34 (21.3%), 62 (38.8%), 18 (11.3%), 24 (15%), and 22 (13.8%), respectively. Median WBC count, RBC count, hemoglobin, platelets, MCV, MPV, PDW, AST, ALT and GGT were 5.6 × 10^9^/L (4.7, 6.9), 4.7 × 10^12^/L (4.3, 5.1), 146 g/L (130, 157), 175 × 10^9^/L (133, 207.5), 91.1 fL (87.8, 94.1), 11.2 fL (10, 12), 13.2% (11.9, 14.7), 32 IU/L (24, 45.2), 41.7 IU/L (25, 67.6), and 35 IU/L (21, 64.3), respectively. Median APRI, Fib-4, and GPR were 0.49 (0.30, 0.97), 1.16 (0.86, 1.93), and 0.39 (0.24, 0.73), respectively.  Table 2 displays median values of some laboratory parameters and serum markers, which were categorized by different METAVIR fibrosis stages. Subjects with significant fibrosis (≥F2) had higher age (39.5 (32.0, 48.0) versus 34.5 (29.0, 42.0), *P* = 0.011), AST (32.7 (24.8, 52.3) versus 25.5 (20.5, 36.3), *P* = 0.002), ALP (73 (58.5, 96.1) versus 57.5 (44.8, 65), *P* = 0.01), and GGT (35.9 (20.9, 72.0) versus 18.5 (13.8, 42.8), *P* = 0.01) compared with patients without significant fibrosis (Table 3).

**Figure 1:**
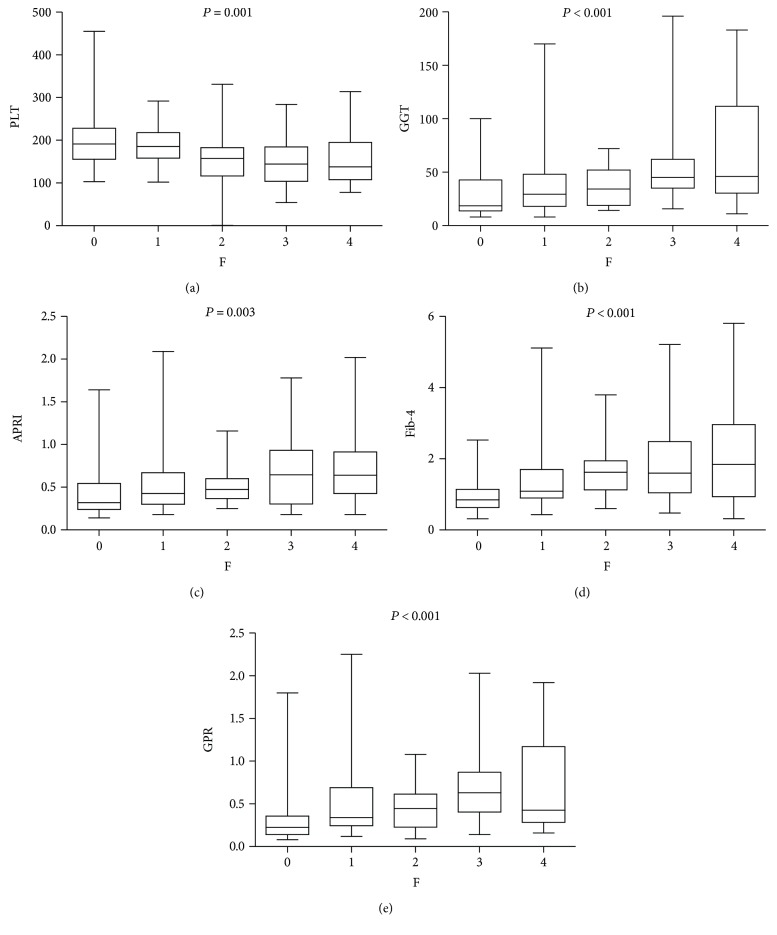
Box plots of platelet (a), gamma-glutamyl transpeptidase (GGT) (b), aspartate transaminase-to-platelet ratio index (APRI) (c), fib-4 (d), and GGT-to-platelet ratio (GPR) (e) according to METAVIR fibrosis stages.

Box plots of GGT, platelet, GPR, APRI, and Fib-4 in relation to the METAVIR fibrosis stage are displayed in Figures 1(a), 1(b), 1(c), 1(d), and 1(e). As shown, the GGT values had a positive correlation with METAVIR scores, while platelet values had a negative one. The newly applied index, GPR, was statistically positively correlated with METAVIR fibrosis stages.

**Figure 2:**
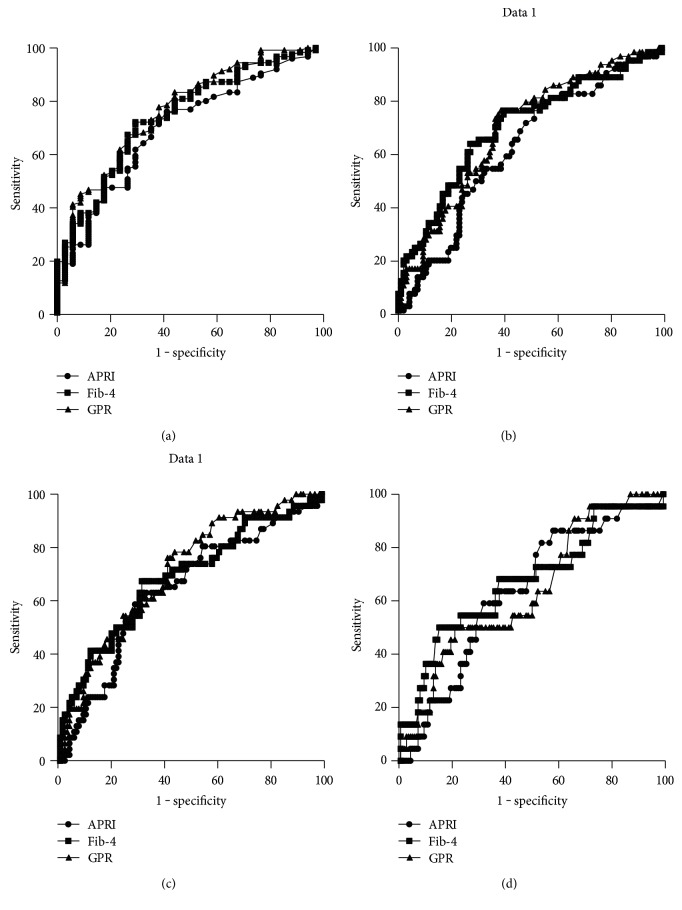
ROC curves of GPR, APRI, and Fib-4 for diagnosing fibrosis (a), significant fibrosis (b), extensive fibrosis (c), and cirrhosis (d).

The diagnostic performances of GPR, APRI, and Fib-4 for diagnosing fibrosis and cirrhosis were shown in Table 4 and Figure 2. The AUROCs of GPR for fibrosis (0.77 versus 0.70, *P* = 0.03), significant fibrosis (0.70 versus 0.63, *P* = 0.02), and extensive fibrosis (0.71 versus 0.64, *P* = 0.02) were significantly higher than those of APRI. The AUROCs of GPR and Fib-4 for fibrosis (0.77 versus 0.75, *P* = 0.14), significant fibrosis (0.70 versus 0.70, *P* = 0.22), extensive fibrosis (0.71 versus 0.68, *P* = 0.13), and cirrhosis (0.64 versus 0.67, *P* = 0.24) were comparable. The diagnostic thresholds and accuracies of serum models were shown in Table 5. Notably, sensitivity, specificity, positive predictive value, and negative predictive value of GPR were 77%, 64%, 59%, and 80%, respectively, for significant fibrosis and 81%, 50%, 28%, and 91%, respectively, for cirrhosis.

## 4. Discussion

Nowadays, lots of novel noninvasive markers or models have been proposed to predict the histologic severity of liver fibrosis, which include APRI, Fib-4, and especially transient elastography. Transient elastography (Fibroscan) is a quick and reproducible method to measure liver stiffness. Its operating principle is sonographic measurement. The propagation velocity of an elastic wave is induced by the device, and the detected wave speed reflects the medium stiffness. Fibroscan acts well in detecting fibrosis, but it is still expensive for developing regions or areas [9–17]. Notably, in June 2015, Lemoine et al. reported a new serum fibrosis marker, the GPR for CHB patients, to predict significant fibrosis and cirrhosis in West Africa. They compared GPR with APRI and Fib-4 in three cohorts (Africa and Europe) [5]. In 2016, two Chinese research groups independently performed studies to evaluate the diagnostic accuracy of GPR both in Shanghai, China, and in Hebei, China [7, 8]. However, the results of these 3 research teams were conflicting, and all of them expected further data for the future [5, 7, 8]. In our retrospective study, we found that GPR showed advantages than APRI in diagnosing fibrosis, significant fibrosis, and extensive fibrosis and it was comparable with Fib-4 in diagnostic performances.

There may be some explanations for different results of all these four studies. Firstly, based on the present epidemiological evidence, HBV genotype A is highly prevalent in sub-Saharan Africa, Western Africa, and Northern Europe, while genotypes B and C are commonly seen in Asia [18–20]. Furthermore, in China, genotype C was the overwhelming majority in northeastern regions. But in eastern regions, genotype C is dominant, followed by genotype B [21]. The giant differences in HBV genotypes may be one reason why the results were so variant. Secondly, in Lemoine et al.'s cohorts [5], most patients are HBeAg seronegative and had low HBV DNA levels, while the three retrospective studies (Li et al.'s [7], Wang et al.'s [8], and ours) in China mostly covered patients being HBeAg seropositive and having high HBV DNA levels. This may also lead us to different conclusions. Thirdly, our study adopted the same histological scoring system as the Western African cohorts did the METAVIR scoring system, while the Shanghai and Hebei cohorts chose different systems, which were the Scheuer scoring system and 2000 Xi'an Viral Hepatitis Management Guidelines. Fourthly, there were differences in sample size and spectrum bias of fibrosis stages, which could lead to different results between these 4 studies. The sample size of African cohorts and ours is relatively small, and few patients were in cirrhosis stage.

There is no doubt that some limitations in our study should be realized. Firstly, our study were part of a single-center trial, and our patients were retrospectively enrolled, which means this novel GPR model has not been validated yet by large-sample prospective studies. Secondly, since we cannot invite all CHB patients for liver biopsy, patients included may not be representative of the general CHB subjects in China and the sensitivities could be somewhat overestimated. The uneven distribution of fibrosis within the studied population with low prevalence of advanced fibrosis/cirrhosis (40%) represents a spectrum bias. Thirdly, due to the study design and the lack of enough clinical materials, we had not set any forms of cohorts, and inevitably, the lack of a validation cohort undermines the scientific power of our study.

Only a few studies have been carried out to report the diagnosing accuracy of GPR in China, especially in the north-east areas, so our study surely got some importance and highlights. Firstly, the north-east regions are not so well developed as the east areas of China, let alone the developed countries. So the GPR, which is based on parameters of routine blood tests, means a lot for the management of CHB patients in resource-limited settings due to its cost, convenience, and relative sensitivity. Secondly, since our results are different to the Shanghai cohorts' and Hebei cohorts', it may capture more interest and involve more scientists in this direction, which could be more meaningful for us. We hope that Chinese medical workers could cooperate and perform more studies (both retrospective and prospective ones) to evaluate these current noninvasive models for diagnosing cirrhosis and create new ones as well.

In conclusion, our findings indicate that GPR could be a simple and useful serum model for the prediction of fibrosis in CHB patients in China.

## Figures and Tables

**Table 1:** Baseline characteristics of the study population.

Variables	Total (*n* = 160)
*Demographic*	
Age (years)	39 (31.2, 47)
Male	101 (63.13%)
*Hepatitis virus*	
HBeAg positivity	76 (47.5%)
Detectable HBV DNA	150 (93.75%)
HBV DNA (IU/mL)	11200 (445.25, 1056100)
*Hematological parameters*	41.7 (25, 67.6)
WBC count (109/L)	5.6 (4.7, 6.9)
RBC count (1012/L)	4.7 (4.3, 5.1)
Hemoglobin (g/L)	146 (130, 157)
MCV (fL)	91.1 (87.8, 94.1)
Platelet count (109/L)	175 (133, 207.5)
PDW (%)	13.2 (11.9, 14.7)
MPV (fL)	11.2 (10, 12)
*Liver function markers*	
AST (8–40 IU/L)	32 (24, 45.2)
ALT (8–40 IU/L)	41.7 (25, 67.6)
GGT (5–54 IU/L)	35 (21, 64.3)
*Fibrosis markers*	
GPR	0.39 (0.24, 0.73)
APRI	0.49 (0.30, 0.97)
Fib-4	1.16 (0.86, 1.93)
*Histological (METAVIR scores)*	
0	34 (21.3%)
1	62 (38.8%)
2	18 (11.3%)
3	24 (15%)
4	22 (13.8%)

**Table 2:** Laboratory parameters and serum markers of different stages.

Stage	F0	F1	F2	F3	F4	*P* value
Age	34.5 (29, 42)	38.5 (32, 48)	44.5 (38.5, 51)	39 (32, 47.75)	38.5 (31.5, 47.5)	0.075
AST	25.5 (20.5, 36.25)	31.95 (24.75, 85.75)	31.15 (24.6, 37.18)	39.45 (24.52, 52.5)	34.5 (27, 76.9)	0.022
ALT	34 (22.5, 54.75)	47.3 (30.08, 96.08)	35.45 (27.78, 50.5)	49.5 (23.25, 66.05)	32.5 (24.75, 59.5)	0.238
*γ*-GT	18.5 (13.75, 42.75)	34.5 (22.9, 72.85)	34.65 (19.42, 53.83)	47 (35.4, 68)	46.5 (30.6, 126.05)	<0.001
PLT	191.5 (155.75, 228.25)	185.5 (158.25, 217.5)	157.5 (116.25, 182.5)	144.5 (104, 184.25)	137.5 (107.75, 195)	0.001
HBV DNA	984 (112.5, 14825000)	162500 (819.75, 5130000)	806 (364.75, 33475)	17800 (877.75, 484500)	11750 (834.75, 108375)	0.146
GPR	0.19 (0.15, 0.29)	0.34 (0.25, 0.70)	0.47 (0.25, 0.72)	0.63 (0.41, 1.27)	0.60 (0.30, 1.24)	<0.001
APRI	0.21 (0.15, 0.35)	0.49 (0.31, 1.24)	0.49 (0.38, 0.85)	0.73 (0.35, 1.60)	0.74 (0.44, 1.40)	0.003
Fib-4	0.84 (0.63, 1.14)	1.09 (0.90, 1.70)	1.63 (1.13, 1.96)	1.59 (1.05, 2.48)	2.03 (0.95, 3.18)	<0.001

**Table 3:** Analysis of factors associated with the presence of fibrosis.

Factors	HBV (*n* = 160)		
	No fibrosis	Fibrosis	*P* value
	*n* = 34	*n* = 126	
Age	34.5 (29, 42)	39.5 (32.0, 48.0)	0.011
AST	25.5 (20.5, 36.3)	32.7 (24.8, 52.3)	0.002
ALT	34 (22.5, 54.8)	43.4 (25.0, 68.5)	0.92
ALP	57.5 (44.8, 65)	73 (58.5, 96.1)	0.01
GGT	18.5 (13.8, 42.8)	35.9 (20.9, 72.0)	0.01
PLT	191.5 (155.8, 228.3)	172 (131.8, 202.5)	0.42

**Table 4:** Diagnostic performances of serum models for liver fibrosis and cirrhosis.

	Fibrosis (≥F1)			Significant fibrosis (≥F2)			Severe fibrosis (≥F3)			Cirrhosis (F4)		
	AUROC	(95% CI)	*P* value	AUROC	(95% CI)	*P* value	AUROC	(95% CI)	*P* value	AUROC	(95% CI)	*P* value
APRI	0.7	0.61–0.80	<0.001	0.63	0.54–0.72	0.006	0.64	0.55–0.74	0.006	0.63	0.52–0.78	0.043
GPR	0.77	0.68–0.86	<0.001	0.7	0.62–0.78	<0.001	0.71	0.62–0.79	<0.001	0.64	0.56–0.80	0.036
Fib-4	0.75	0.66–0.84	<0.001	0.7	0.62–0.79	<0.001	0.68	0.59–0.78	<0.001	0.67	0.52–0.81	0.009
Comparison of AUROC												
GPR and APRI	0.03			0.02			0.02			0.25		
GPR and Fib-4	0.14			0.22			0.13			0.24		
APRI and Fib-4	0.06			0.01			0.07			0.23		

APRI = aspartate transaminase-to-platelet ratio index; AUROC = the area under the receiver operating characteristic curve; CI = confidence interval; Fib-4 = fibrosis index based on the four factors; GPR = gamma-glutamyl transpeptidase-to-platelet ratio index.

**Table 5:** Diagnostic thresholds and accuracies of serum models for liver fibrosis and cirrhosis.

	Cut-off value	Se (%)	Sp (%)	PPV (%)	NPV (%)
GPR					
≥F1	0.26	80	65	88	43
≥F2	0.35	77	64	59	80
≥F3	0.38	76	60	48	86
≥F4	0.72	81	50	28	91
APRI					
≥F2	0.5	61	52	49	62
	1.5	20	86	50	62
≥F4	1	27	77	16	87
	2	23	88	25	88
Fib-4					
≥F3	1.45	59	69	44	81
	3.25	22	95	63	75

APRI = aspartate transaminase-to-platelet ratio index; AUROC = the area under the receiver operating characteristic curve; CI = confidence interval; Fib-4 = fibrosis index based on the four factors; GPR = gamma-glutamyl transpeptidase-to-platelet ratio index; NPV = negative predictive value; PPV = positive predictive value; Se = sensitivity; Sp = specificity; WHO = World Health Organization.

## Abbreviations

AUROC: Area under the ROC curve
ALP: Alkaline phosphatase
ALT: Alanine transaminase
APRI: Aspartate transaminase-to-platelet ratio index
AST: Aspartate aminotransferase
CHB: Chronic hepatitis B
CI: Confidence interval
Fib-4: Fibrosis index based on 4 factors
GGT: Gamma-glutamyl transpeptidase
GPR: Gamma-glutamyl transpeptidase-to-platelet ratio
HBV: Hepatitis B virus
HCC: Hepatocellular carcinoma
IQR: Interquartile range
LB: Liver biopsy
MCV: Mean corpuscular volume
MPV: Mean platelet volume
PDW: Platelet distribution width
RBC: Red blood cell
ROC: Receiver operating characteristic
WBC: White blood cell.
